# PRP Treatment Efficacy for Tendinopathy: A Review of Basic Science Studies

**DOI:** 10.1155/2016/9103792

**Published:** 2016-08-16

**Authors:** Yiqin Zhou, James H-C. Wang

**Affiliations:** ^1^MechanoBiology Laboratory, Department of Orthopaedic Surgery, University of Pittsburgh School of Medicine, 210 Lothrop Street, BST, E1640, Pittsburgh, PA 15213, USA; ^2^Joint Surgery and Sports Medicine Department, Shanghai Changzheng Hospital, Second Military Medical University, Shanghai 200003, China

## Abstract

Platelet-Rich Plasma (PRP) has been widely used in orthopaedic surgery and sport medicine to treat tendon injuries. However, the efficacy of PRP treatment for tendinopathy is controversial. This paper focuses on reviewing the basic science studies on PRP performed under well-controlled conditions. Both* in vitro *and* in vivo *studies describe PRP's anabolic and anti-inflammatory effects on tendons. While some clinical trials support these findings, others refute them. In this review, we discuss the effectiveness of PRP to treat tendon injuries with evidence presented in basic science studies and the potential reasons for the controversial results in clinical trials. Finally, we comment on the approaches that may be required to improve the efficacy of PRP treatment for tendinopathy.

## 1. Introduction

Tendons are dense connective tissues that link muscles to bones. Thus, they transmit muscular forces to bones and enable joint movements. As a consequence, tendons are subject to large mechanical loads that may cause injuries and affect tendon function. In orthopaedic clinics, tendon and ligament injuries are one of the most prevalent health problems with about 16.4 million individuals seeking medical intervention every year in the United States [1, 2]. Among the tendons, Achilles, patellar, rotator cuff, and forearm extensor tendons are the easiest to injure due to overuse [3]. Typically, tendons are hypovascular. However, histopathological observations of chronic tendon injury or tendinopathy have revealed hypervascularity and disorganization in vessel distribution, which may affect the mechanical properties of tendons and induce pain. Tendinopathic tendons also feature interstitial gaps (microtears), discontinuous collagen fibers, and a number of degenerative changes including lipid deposition, proteoglycan accumulation, and calcification [2]. They also have decreased total collagen content, increased collagen type III/collagen type I ratio, increased expression of matrix metalloproteinases (MMPs), MMP-1, MMP-3, and MMP-9, and decreased expression of the MMP inhibitors [4, 5]. Apart from changes in tendon metabolism, high inflammation has been also reported at the microinjury sites that damage the tendon tissue if left untreated [6–8].

In recent years, a popular option for treating musculoskeletal injuries including tendinopathy is Platelet-Rich Plasma (PRP) [9–11]. Currently PRP is administered to almost 86,000 athletes in the United States and Europe to treat acute and chronic tendon, ligament, and muscle injuries [12]. Because of its wide spread use, it is estimated that the market value of PRP will reach $126 million this year [13]. The key components of PRP are the platelets, which are anucleate cytoplasmic fragments produced by megakaryocytes in the bone marrow [14]. Platelets have long been recognized to maintain tissue hemostasis. But accumulating evidence indicates that platelets may have a much wider role in tissue healing because they store and release a wide range of bioactive factors including growth factors (e.g., TGF-*β* and HGF). These factors are secreted by the dense granules, *α*-granules, and lysosomes in platelets [15]. Apart from platelets, PRP also has other components such as plasma, leukocytes, neutrophils, and monocytes with some leukocyte-containing PRP (L-PRP) containing residual erythrocytes, which also contain and/or release some bioactive factors. Plasma contains proteins such as albumin, globulins, fibrinogen, complement, and clotting factors and electrolytes, hormones, and biomarkers. The key components of leukocytes are neutrophils and monocytes, which may also release many bioactive factors and proteins. Neutrophils mainly release myeloperoxidase, bactericidal phagocytins, collagenase, gelatinase, and proteases. Monocytes secrete platelet-activating factor, TGF-*β*, VEGF, FGF, and EGF [16]. Many of these factors have been shown to influence tendon healing [17, 18].

The main advantages of PRP that enable its wide spread use are its safety and simple preparation and administration methods. PRP is safe because it is an autologous product derived from a patients' own blood and contains platelets and bioactive factors that can modify the biological microenvironment at injury sites, thus enhancing tissue healing. More importantly, PRP is not known to have any adverse effects unlike the commonly used nonsteroid anti-inflammatory drugs (NSAIDs) that are known to affect the gastrointestinal, cardiovascular, and renal systems [19, 20]. PRP is easy to use because of the simple preparation protocols; in fact, a number of PRP preparation kits are commercially available that are widely used in orthopaedic clinics. Besides, the application of PRP in the form of injections is also noninvasive and allows easy administration in clinical settings.

Thus far, numerous basic science studies have shown the beneficial effects of PRP in healing tendon injuries. Specifically, growth factors in PRP have significantly enhanced the healing of tendon injuries such as tendinopathy [21–24]. These include PDGF, TGF-*β*, VEGF, EGF, IGF-I, FGF, and HGF [24–26]. When platelets are activated, not only are the growth factors released, but PRP also forms a fibrin gel, which provides a conducive bioscaffold for migrating cells thus incorporating new cells for tendon healing [27]. Therefore, PRP can be regarded as a promising treatment option for tendinopathy. However, the efficacy of PRP to treat tendinopathy in clinical trials is still controversial. Therefore, in this review we discuss evidence for the use of PRP presented in basic science studies conducted* in vitro* and* in vivo *under well-controlled conditions to determine the basis of PRP applications in clinical settings.

## 2. Basic Science Studies on the PRP Treatment of Tendinopathy

### 2.1. *In Vitro* Studies

#### 2.1.1. Cell Proliferation

First, PRP has been reported to induce the proliferation of the two tendon cell types, tenocytes and tendon stem/progenitor cells (TSCs). Tenocytes are the predominant cells in tendons and are essential to maintain tendon homeostasis. When tendons are injured, tenocytes proliferate and help in tendon repair; however, the proliferation rate of tenocytes is limited. A number of studies have shown that PRP can promote tenocyte proliferation* in vitro *[28, 29]. Wang et al. demonstrated that treatment of human tenocytes with platelet-rich clot releasate (PRCR) accelerated their proliferation in a dose-dependent manner [30]. Besides tenocytes, tendons also contain TSCs, which are tendon specific adult stem cells that make up to ~5% of the tendon cell population. TSCs were discovered in recent years in the tendons of mice, rats, rabbits, and humans [31–34]. TSCs have a high proliferation rate and PRCR has been reported to enhance it further [35]. In addition, PRP is also known to accelerate the proliferation rates of circulating stem cells such as bone marrow stem cells (BMSCs) and adipose derived stem cells (ADSCs) thereby accelerating tendon healing [28, 36, 37]. Therefore, ample evidence suggests that PRP has the ability to promote tendon cell proliferation. However, the optimal platelet concentration or the PRP composition needed for an effective treatment of tendinopathy is not clear [29]. Previous studies presumed that the effect of PRP may positively correlate with its concentration [28, 36, 37]. However, the “more is better” theory is not supported by studies because PRP-induced cell proliferation increased in a dose-dependent manner only up to a certain concentration. Specifically, the concentration of platelets from human PRP strongly induced tenocyte proliferation and migration only up to 1 × 10^6^ platelet/*μ*L concentration but above that cell proliferation and migration were reduced [38]. Moreover, the effect of rabbit PRP on cell proliferation* in vitro *also diminished when the concentration of PRP was 20% or more than the amount of the culture medium [39]. Interestingly, leukocytes in PRP, widely known for their deleterious effects, did not inhibit tendon cell proliferation but increased it in comparison to PRP containing no leukocytes [39].

#### 2.1.2. Tenocyte Differentiation

A number of studies have demonstrated the ability of PRP to induce TSCs differentiation into tenocytes. PRCR was shown to induce rabbit TSCs to differentiate into tenocytes* in vitro*, and the newly formed tenocytes were *α*-SMA positive indicating that they were active tenocytes [35, 39]. One study specifically showed that PDGF in PRP promoted tenogenic differentiation of ADSCs [36]. It should be noted that currently there are no known tenocyte specific markers, and tenocyte differentiation is assessed by using a panel of markers including collagen types I and III and tenomodulin. Therefore, there is uncertainty regarding whether these differentiated cells are true tenocytes or tendon progenitor cells that have differentiated from TSCs to a certain extent. More importantly, PRCR treatment of TSCs did not induce the expression of non-tenocyte-related genes (PPAR*γ*, Sox-9, and Runx-2) suggesting their safety in tendon injury treatments [35]. Autologous PRCR was also shown to inhibit the differentiation of rat TSCs towards nontenocyte lineages in a PRP dose-dependent manner [21]. Along these lines, an important finding was made by Zhang and Wang who reported that PRCR could only induce TSCs to differentiate into tenocytes but cannot reverse nontenocyte differentiation that is well underway in advanced stage tendinopathic tendons [40]. This finding may explain why PRP injection to treat advanced stage tendinopathy, where nontendinous tissues are predominant in the lesion sites, may not be effective in clinics. Such advanced stage tendinopathy may be effectively repaired by removing the tendon lesions by tissue debridement followed by PRP treatments. In this case, removal of the nontenogenic materials in the tendon that may negatively impact PRP effects may result in an improvement in the PRP treatment efficacy.

#### 2.1.3. Anabolic Effects

A number of* in vitro* studies have shown that PRP can influence the metabolism of tendon cells involved in the wound healing process. PRP was shown to increase total collagen synthesis in both tenocytes and TSCs [35, 39] and specifically enhance the gene expression of collagen types I and III [41]. An* in vitro* study on tendon tissues used PRP to treat horse flexor digitorum superficialis tendon and found that PRP treatment increased the expression of collagen types I and III and collagen type I/collagen type III ratio. However, in another cell culture study, PRP treatment did not significantly change the ratio of collagen type I/collagen type III although the gene expression of collagen types I and III significantly increased [42].

PRP not only affects collagen but also affects the expression of other tendon related genes and proteins. For example, PRP treatment increased the mRNA and protein expression of tenocyte-related genes (scleraxis (SCX) and tenascin-C) by activating the focal adhesion kinase (FAK) and extracellular-regulated kinase (ERK) 1/2 signaling pathways [43]. Furthermore, PRP treatment has been shown to enhance the expression of COMP, decorin, and tenascin-C [35, 42–46]. COMP is a tendon healing related glycoprotein, which is abundant in the normal tendon but is depleted in fibrous scar tissue. Decorin is a matrix proteoglycan and is also abundant in the tendon and plays a role in matrix assembly by binding to collagen type I fibrils. Tenascin-C is also a glycoprotein abundant in developing tendons. These noncollagenous matrix markers are the indicators of matrix synthesis, and an increase in their expression by PRP treatment also signifies the beneficial anabolic effects of PRP on tendons.

However, PRP's positive influence on the anabolism of tendon cells has not been consistent in studies. One reason for this is the presence or absence of leukocytes in PRP. Based on the amounts of leukocytes, PRP may be classified as pure-PRP (P-PRP) containing a few or no leukocytes and L-PRP that contains more leukocytes. Since the protocols for preparing PRP vary in basic science studies and clinical trials, variations in PRP treatment effects should be expected. An* in vitro* study showed that PRP could stimulate human tenocyte proliferation and total collagen production, but it decreased the gene expression of collagen types I and III without affecting the collagen I/collagen III ratio. It should be noted that in this study PRP contained high levels of leukocytes [44]. In another study, increasing the platelet concentration in L-PRP significantly reduced collagen type 1A1 and collagen type IIIA1 synthesis in tendons, despite the delivery of more anabolic growth factors [47]. In yet another* in vitro* study, both L-PRP and P-PRP without leukocytes were found to enhance the gene and protein expression of collagen types I and III but L-PRP induced higher collagen type III and lower collagen type 1 expression than P-PRP [39]. Since a high collagen type III/collagen type I ratio indicates fibrosis that reduces the mechanical strength of tendons, it appears that administering L-PRP, especially when leukocyte levels are high, may lead to inferior outcomes in tendon healing. However, platelets and leukocytes had differential effects on the collagen ratio. While platelets positively influenced collagen type I and thereby increased collagen type I/collagen type III ratio, leukocytes increased the amount of collagen type III thereby negatively influencing the collagen type I/collagen type III ratio [45]. Furthermore, platelets decreased collagen type III levels but increased COMP and decorin while leukocytes had the opposite effects [45]. Together, these studies indicate clearly that leukocytes in PRP may negatively affect the anabolic effects of PRP and may lead to scar formation by increasing the collagen type III/collagen type I ratio [48].

#### 2.1.4. Catabolic Effects

Pure-PRP has not been implicated in much catabolic activities while L-PRP is known to induce catabolic effects. Treatment of tenocytes with PRP, likely P-PRP, did not influence the catabolic molecules, MMP-3 and MMP-13 [41]. In fact, the expression of MMP-13 in tenocytes decreased after PRP treatment* in vitro*, and the platelet concentration negatively correlated with MMP-3 and MMP-13 levels [46]. However, PRCR upregulated MMP-1 and MMP-3 expression [44] likely because of the presence of large amounts of leukocytes in PRCR. In a recent study, we showed that L-PRP significantly induced the expression of MMP-1 and MMP-13 while P-PRP only slightly increased the expression of MMP-1 when compared to the control [39]. These studies clearly indicate that leukocytes in PRP are the key factors that induce catabolic actions in tendons/tendon cells.

#### 2.1.5. Anti-Inflammation

PRP also plays an active role in the inflammatory aspects of tissue healing. It was shown that PRP treatment of tendon cells* in vitro *could induce the release of HGF, which is a major anti-inflammatory growth factor [49]. Results from a recent study were also consistent with these findings and reported that PRP treatment increased VEGF and HGF expression in tendinopathic tendons [50]. Furthermore, the anti-inflammatory effect of HGF was shown by the reduction in the levels of COX-1, COX-2, and PGE_2_, the proinflammatory mediators in tendon cells treated with PRP or HGF. However, addition of HGF antibodies to the* in vitro* culture overruled this suppression suggesting that HGF plays a crucial role as an anti-inflammatory mediator in PRP preparations [49]. Similarly, injection of HGF antibodies into wounded mouse Achilles tendons reversed the reduction in PGE_2_, COX-1, and COX-2 protein levels caused by PRP or HGF injections [49]. Additionally, HGF in PRP increased the expression of I*κ*B*α*, which is an inhibitor of NF*κ*B [51] and a well-known regulator of immune response to infection. This is highly relevant in tendinopathy treatments because NF*κ*B expression is upregulated in the tenocytes of tendons with inflammation [52] and therefore can be reduced after PRP treatment. Potentially, IGF-1 or PDGF in PRP can also inhibit I*κ*B kinase *α* (IKK*α*) and suppress the production of NF*κ*B thereby inhibiting inflammation [53]. In addition, it was shown that PRP treatment could decrease the expression of the proinflammatory cytokine, IL-6, and its ligand, CXCL-6, and IL-8 along with its ligand, CXCL-8, in tendon cells isolated from tendinopathic tendons [50]. Such reduction was shown to be effected specifically by HGF treatment that decreased the production of IL-6 and increased the anti-inflammatory cytokine, IL-10, levels [54]. TGF-*β*, also an anti-inflammatory cytokine present in PRP, has been also shown to control local inflammation [55].

However, leukocytes in PRP could potentially increase inflammation because they significantly increased the gene and protein expression of IL-1*β*, IL-6, and TNF-*α* in tendon cells [39]. This demonstrates that leukocytes can exacerbate inflammation in tendon cells but P-PRP without leukocytes can be anti-inflammatory because it decreased the gene expression of IL-6 when compared to untreated controls [39]. Similarly, PRP also reduced the gene expression and production of IL-6 in tendon cells when compared to cells treated with IL-1*β* [50]. It should be noted that IL-6 levels in tendon cells cultured in 2D and 3D hydrogels were increased by P-PRP [56]; therefore, future study may be still needed to determine the precise effects of P-PRP on IL-6. McCarrel et al. [45, 57] also showed that high concentration of leukocytes in PRP could induce higher expression of IL-1*β* and TNF-*α* when compared to P-PRP. Because platelets recruit leukocytes and progenitor cells to the sites of vascular injury and inflammation, they induce changes in cell permeability and promote chemotaxis and cell proliferation, which are essential steps in tissue repair. Macrophage, a kind of leukocyte, was reported to be involved in the maintenance of inflammatory state or innate immune response. It also induced tenocytes to synthesize relevant amounts of MCP-1/CCL2 and RANTES/CCL5, which could mediate migration of more monocytes/macrophages that can trigger inflammatory and angiogenic mechanisms [56, 58]. L-PRP can be directly involved in the inflammatory response by producing and releasing inflammatory mediators such as the cytokines, IL-1*β* and CD40L, and chemokines, CXCL1 and CCL2. Besides, PRP induces the expression of chemokine receptors, particularly CCR1, CCR3, CCR4, and CXCR4, thus regulating the inflammatory response associated with the healing process [55].

#### 2.1.6. Antibiotic Effects

Furthermore, platelets are a source of active metabolites and proteins that promote heterotypic cell interactions, provide a surface together with cell-derived microparticles that promote coagulation and protease activation, and play an active role in sepsis and fighting infection (including promoting the innate immune response). Intravia et al. [59] compared the antibiotic effect of two different PRP preparations: PRPLP with lower leukocyte and platelet concentrations and PRPHP with higher leukocyte and platelet concentrations. The results showed that both PRP preparations significantly decreased bacterial (*MRSA*,* P. acnes, S. epidermidis*, and* S. aureus*) growth compared to whole blood but no difference in antibacterial activity was observed between the two products. This indicates not only the antibiotic effect of PRP but also that the leukocyte and platelet concentrations may not influence the antibiotic effects of PRP.

Based on these* in vitro* studies, it is evident that PRP can enhance cell proliferation and also induce the tenocyte differentiation of stem cells thus replenishing cell resources for effective tendon healing. PRP also induces anabolic effects and increases collagen production thereby helping extracellular matrix restoration and tissue remodeling in healing tendons. In addition to platelets, plasma in PRP has been found to positively influence cell attachment and spreading on the fibrin scaffold, as well as promoting cell proliferation [60]. However, as shown above, platelet and leukocyte concentrations could significantly affect PRP function. Too high or too low platelet concentration is not advisable for the clinical treatment of tendinopathy with PRP. As discussed, L-PRP may cause higher catabolic and inflammatory actions than P-PRP indicating that leukocytes play a major role in reducing the efficacy of PRP.

*PRP versus Steroids*. Lastly, current clinical practices commonly use corticosteroids to treat tendinopathy although they are known to cause multiple side effects. Application of dexamethasone, a corticosteroid, to hTSCs in culture induced nontenocyte differentiation visualized by a change in the cell shape, a near-complete suppression of collagen type I expression, and upregulation of non-tenocyte-related genes (PPAR*γ* and Sox-9) especially at higher concentrations (>10 nM) [61]. Another study showed that dexamethasone reduced cell viability and increased the number of senescent cells. However, after cotreatment with 10% PRP, dexamethasone-induced senescence was markedly reduced [62]. Similarly, exposure to methylprednisolone alone decreased human tenocyte viability, but the addition of PRP partially reversed this negative effect [63]. Moreover, incorporation of corticosteroids with PRP injection was shown to compromise the potential beneficial effects of PRP on tendon cells with reduced cell viability at the site of tendon injury [64]. Thus, PRP may be a better alternative to treat tendinopathy than steroid treatments.

### 2.2. *In Vivo* Studies

PRP has been shown to treat tendinopathy and promote tendon healing positively in* in vivo* animal studies. Intratendinous injections of PRP to treat tendinopathy in rat patellar and Achilles tendons increased joint mobilization and improved tendon fiber organization 25 days after treatment [65]. In this study, the authors also tested the toxicity of PRP by injecting it into normal tendons and found no difference in tested parameters between the control and PRP treated normal tendon thus showing that PRP poses no threats for its use* in vivo *[65]. Spang et al. [66] compared the effect of platelet concentrate and saline in a rat tendon healing model* in vivo *and found that at 14 days after treatment ultimate tensile load and energy absorbed to failure increased significantly in the platelet concentrates treated rats when compared to the saline treated rats. Histological results also showed more elongated cells indicating the presence of tenocytes and the absence of chondrocytes in the platelet concentrate treated group thus confirming the safety of PRP use* in vivo*. Lyras et al. [67] observed the effect of PRP gel in the early phase of patellar tendon healing and found that after 2 weeks PRP treatment increased load at failure by 72.2%, ultimate stress by 39.1%, and stiffness by 53.1% compared to untreated controls. Immunohistochemical analysis during the first two weeks of healing after PRP treatment further showed significant increase in blood vessel density in comparison with the control. Moreover, angiogenesis also significantly decreased in the PRP group 3 and 4 weeks after treatment [68]. PRP treatment also induced better cell orientation and tissue maturation. These results indicated that PRP could accelerate the tendon wound healing process. After a longer healing period, another study reported fewer neovessels in tendons after PRP treatment [65]. Lastly, PRP was also found to increase the expression of growth factors (IGF-I) in healed tendons [69].

Recent biologics approaches have also combined PRP with other tissue engineering modalities to enhance tendon healing. Particularly, stem cells in conjunction with PRP have shown promising results in the treatment of tendinopathy. PRP + BMSC treatment of tendon wounds in dogs significantly increased the strength and stiffness of healing tendons compared to the groups that used either of them separately [37]. Carvalho et al. performed an animal RCT (Randomized Controlled Trial) and found that, after 16 weeks, the combination of ADMSCs and platelet concentrate prevented the progression of lesion, induced a greater organization of collagen fibers, and decreased inflammatory infiltrates [70]. Furthermore, the combined use of TSCs and PRP showed synergistic effects (higher collagen I mRNA level) on tendon healing [43, 71]. However, injection of PB-MSCs (Peripheral Blood-Derived Mesenchymal Stromal Cells) and PRP together into injured sheep digital flexor tendons did not provide an additional benefit when compared to treatment with PB-MSCs alone [72].

Other modalities have also been used along with PRP to treat tendon injuries. Moshiri et al. reported that the use of platelet gel embedded within a 3D collagen implant in an Achilles tendon defect in rabbits was effective in healing, modeling, and remodeling the tendon [73]. Besides, Barbosa et al. reported that low-level laser therapy combined with PRP increased the deposition of collagen type I and enhanced regeneration of the tendon tissue [74].

Thus, with an exception of a few, most* in vivo* animal studies demonstrated that PRP treatment can enhance the healing of tendinopathic tendons.

## 3. Concluding Remarks

Tendinopathy is a highly prevalent tendon disorder and plagues a range of individuals from the common person to elite athletes. It may cause extreme pain and affect tendon function, which can impair normal life activities. Because the mechanisms of tendinopathy are not completely understood, the current treatment options for this tendon disease remain largely palliative. PRP is a popular cell-free therapy that is used worldwide to treat tendinopathy. Basic science studies have consistently shown the beneficial effects of PRP on tendons including increased tendon cell proliferation, increased expression of anabolic genes and proteins, and reduced tendon inflammation. However, the efficacy of PRP in clinical trials is not consistent leading to the controversies regarding the PRP treatment efficacy. Among clinical studies, RCTs are considered to be the gold standard in assessing the efficacy of PRP treatments in clinical settings. However, when an RCT study yields negative results on PRP treatment of tendinopathy, the reasons for the negative results should be carefully analyzed. A number of factors could cause the negative results in RCTs. The most common is the relatively small sample size. Considering the fact that the extremely variable responses of humans to any treatment are unavoidable, the use of a small number of subjects in an RCT study will surely reduce the statistical power to detect the treatment effects by PRP. Another major factor in RCTs is the undefined PRP composition in the preparations used in RCT studies. Most clinical studies use PRP, prepared from a commercial kit, and a predetermined dose is administered for all types of tendon injuries and all patients irrespective of age, gender, disease history, and so forth. Basic science studies on the other hand indicate that stem cells could be used to promote tendon wound healing only in early stages but not so effectively in later stages that may be dominated by the presence of degenerative tissues. Therefore, the so-called “one-size-fits-all” approach may be the main reason for the conflicting results observed in the PRP treatment of tendinopathy in clinical studies. Instead of this, we propose the use of an individualized approach based on the conditions of individual patients. Such efforts may improve the efficacy of PRP for the treatment of tendon injuries and may effectively address the controversies on the PRP treatment efficacy in clinical trials.
